# Analysis of urine eosinophils and kidney biopsy findings

**DOI:** 10.1590/2175-8239-JBN-2024-0322en

**Published:** 2026-02-06

**Authors:** Sofia Isabel Leitão e Sousa, Marta Costa Rego

**Affiliations:** 1 Hospital do Divino Espírito Santo de Ponta Delgada, Ponta Delgada, Portugal.; 2 Unidade Local de Saúde de Santo António, Porto, Portugal.

Dear Editor,

Acute interstitial nephritis (AIN) accounts for 10 to 15% of unexplained acute kidney injuries (AKI) in hospitalized patients; the gold standard of diagnosis requires a kidney biopsy with patchy inflammatory infiltrates^1^. In 1978, Galpin et al. suggested the association between eosinophiluria and AIN^2^, but as the number of studies and clinical reports grew, it became apparent that urinary eosinophils (UEs) could be found in many other renal and nonrenal diseases^3^, rendering UEs’ value in AIN diagnosis uncertain. Besides the test’s performance, most studies used experts’ opinion-based diagnosis instead of biopsy proven AIN, reducing diagnosis’ reliability. Despite this evidence, case reports and clinical practice show that eosinophiluria is still used in some centers as an AIN marker, especially when biopsy is not feasible.

To strength the existing evidence, we have conducted a retrospective study of the adult patients who underwent both UEs screening and native kidney biopsy performed in a maximum of seven days’ period at Unidade Local de Saúde de Santo António between 2010 and 2020. We also considered UEs positive screening when >1% of eosinophils were seen in citospin smears of urinary sediment, using Leishman staining. We considered the patients with eosinophiluria screening without biopsy to understand the impact of eosinophiluria in the decision to biopsy. Data was analyzed using Microsoft Excel©, and the chi-square test was used to determine association with a p-value of < 0.01.

We included 1686 patients with eosinophiluria screenings. Of these, 180 (10.7%) had eosinophiluria and 105 (6.2%) underwent native kidney biopsy. Considering the patients that underwent kidney biopsy, 18 had eosinophiluria (17.1%) and 15 had AIN (14.3%). Three patients with eosinophiluria had AIN (16.6 %) (Table 1). The sensitivity of eosinophiluria was 20.0%, the specificity was 83.3%, the positive predictive value (PPV) was 16.7%, and the negative predictive value (NPV) was 86.2%. We found no association between eosinophiluria and the decision to biopsy (p = 0.027).

**Table 1:** Kidney biopsy diagnosis of patients with eosinophiluria

Kidney biopsy diagnosis (N = 18)	N (%)
ANCA vasculitis	4 (22.2)
Amyloidosis	3 (16.7)
**Acute interstitial nephritis**	**3 (16.7)**
IgA nephropathy	2 (11.1)
Membranous nephropathy	2 (11.1)
IgA vasculitis	1 (5.6)
Infection-related glomerulonephritis	1 (5.6)
Class IV lupus nephritis	1 (5.6)
Chronicity findings exclusively	1 (5.6)

Our research supports that eosinophiluria yields low performance for AIN diagnosis and should not be used as its marker at the risk of leading to inappropriate drug withdrawal and steroid’s introduction. Moreover, we found that eosinophiluria had no impact on the decision to biopsy.

Muriithi et al.^4^ published in 2013 the most recent and robust paper on this middleic, including 566 patients with native kidney biopsy. Using a 1% cut-off, eosinophiluria had a 30.8% sensitivity, 68.2% specificity, 15.6% PPV, and 83.7% NPV for AIN. Similarly to us, they found that eosinophiluria was present in a multitude of diagnosis. The lower sensitivity found in our study may be due to the differences in the stain used or in slight variations of kidney biopsy indications.

In conclusion, we believe that almost 50 years after the association between eosinophiluria and AIN was suggested, there is enough evidence to refute it.

When kidney biopsy is not feasible, research into novel biomarkers will likely complement clinical judgment and enrich the decision-making framework, allowing for a safer approach to the diagnosis and treatment of AIN.

## Data Availability

The datasets generated and/or analyzed during the current study are available from the corresponding author upon reasonable request.
