# Supplementary material for: Analysis of urine eosinophils and kidney biopsy findings
Source: J Bras Nefrol. 2026 Feb 6;48(2):e20250322. doi: 10.1590/2175-8239-JBN-2024-0322en (PMC12893768; doi:10.1590/2175-8239-JBN-2024-0322en)
Supplement: Translation

# Translation: Análise de eosinófilos na urina e achados de biópsia renal

**DOI:** 10.1590/2175-8239-JBN-2024-0322pt

**Authors:** Sofia Isabel Leitão e Sousa, Marta Costa Rego

**Affiliations:** 1 Hospital do Divino Espírito Santo de Ponta Delgada, Ponta Delgada, Portugal.; 2 Unidade Local de Saúde de Santo António, Porto, Portugal.

Prezado Editor,

A nefrite intersticial aguda (NIA) é responsável por 10 a 15% dos casos de injúria renal aguda (IRA) de causa desconhecida em pacientes hospitalizados; o padrão-ouro para o diagnóstico requer biópsia renal com infiltrados inflamatórios difusos^1^. Em 1978, Galpin et al. sugeriram a associação entre eosinofilúria e NIA^2^; entretanto, à medida que o número de estudos e relatos clínicos aumentou, tornou-se evidente que os eosinófilos urinários (EU) poderiam ser encontrados em diversas outras doenças renais e não renais^3^, tornando incerto o valor dos EU no diagnóstico da NIA. Além do desempenho do teste, a maioria dos estudos utilizou diagnósticos baseados na opinião de especialistas, em vez de NIA comprovada por biópsia, reduzindo a confiabilidade diagnóstica. Apesar dessas evidências, relatos de casos e a prática clínica demonstram que a eosinofilúria ainda é utilizada em alguns centros como marcador de NIA, especialmente quando a biópsia não é factível.

Para reforçar as evidências existentes, realizamos um estudo retrospectivo com pacientes adultos que foram submetidos tanto à triagem de EU quanto à biópsia renal nativa, executadas em um intervalo máximo de sete dias, na Unidade Local de Saúde de Santo António, entre 2010 e 2020. Também consideramos triagem positiva para EU quando >1% de eosinófilos foram observados em esfregaços de *cytospin* de sedimento urinário, utilizando coloração de *Leishman*. Consideramos os pacientes com triagem para eosinofilúria sem biópsia, a fim de compreender o impacto desse achado na decisão de realizar a biópsia. Os dados foram analisados utilizando o Microsoft Excel© e o teste qui-quadrado foi empregado para determinar associações, com um valor de p < 0,01.

Incluímos 1686 pacientes com exames de eosinofilúria. Destes, 180 (10,7%) apresentaram resultado positivo e 105 (6,2%) foram submetidos à biópsia renal nativa. Considerando os doentes submetidos a biópsia, 18 apresentavam eosinofilúria (17,1%) e 15 aprsentava NIA (14.3%). Três desses pacientes apresentaram NIA (16,6%) (Tabela 1). A sensibilidade da eosinofilúria foi de 20,2%, a especificidade foi de 83,3%, o valor preditivo positivo (VPP) foi de 16,7% e o valor preditivo negativo (VPN) foi de 86,2%. Não foi observada nenhuma associação entre a presença desse marcador e a decisão de realizar biópsia (p = 0,027).

**Tabela 1:** Diagnóstico por biópsia renal em pacientes com eosinofilúria

Diagnóstico por biópsia renal (N = 18)	N (%)
Vasculite associada ao ANCA	4(22,2)
Amiloidose	3 (16,7)
**Nefrite intersticial aguda**	3 (16,7)
Nefropatia por IgA	2 (11,1)
Nefropatia membranosa	2 (11,1)
Vasculite por IgA	1 (5,6)
Glomerulonefrite relacionada à infecção	1 (5,6)
Nefrite lúpica classe IV	1 (5,6)
Achados exclusivamente de cronicidade	1 (5,6)

Nossa pesquisa sustenta que a eosinofilúria apresenta baixo desempenho no diagnóstico de NIA e não deve ser utilizada como marcador, devido ao risco de levar à suspensão inadequada de medicamentos e à introdução indevida de esteroides. Além disso, verificamos que a presença desse achado não teve impacto na decisão de realizar a biópsia.

Muriithi et al.^4^ publicaram, em 2013, o estudo mais recente e robusto sobre esse tema, incluindo 566 pacientes com biópsia renal nativa. Utilizando um ponto de corte de 1%, a eosinofilúria apresentou sensibilidade de 30,8%, especificidade de 68,2%, VPP de 15,6% e VPN de 83,7% para NIA. De forma semelhante ao que observamos, os autores constataram que a eosinofilúria esteve presente em diversos diagnósticos. A menor sensibilidade encontrada em nossa análise pode ser atribuída às diferenças na coloração utilizada ou a variações sutis nas indicações para biópsia renal.

Em conclusão, acreditamos que, quase 50 anos após a sugestão da associação entre eosinofilúria e NIA, há evidências suficientes para refutá-la.

Quando a biópsia renal não é viável, a pesquisa de novos biomarcadores provavelmente complementará o julgamento clínico e enriquecerá a estrutura da tomada de decisão, permitindo uma abordagem mais segura para o diagnóstico e tratamento da NIA.

## Data Availability

Os conjuntos de dados gerados e/ou analisados durante o presente estudo estão disponíveis mediante solicitação razoável ao autor correspondente.
